# Chromosome 16p11.2 deletions: another piece in the genetic puzzle of childhood obesity

**DOI:** 10.1186/1824-7288-36-43

**Published:** 2010-06-11

**Authors:** Laura Perrone, Pierluigi Marzuillo, Anna Grandone, Emanuele Miraglia del Giudice

**Affiliations:** 1 Department of Pediatrics "F. Fede" Seconda Università degli Studi di Napoli, Via Luigi De Crecchio 2, 80138, Napoli, Italy

## Abstract

Ipercaloric diet and reduced physical activity have driven the rise in the prevalence of childhood obesity over a relatively short time interval. Family and twin studies have led to the conclusion that the strong predicitve value of parental body mass index (BMI) mainly stems from genetic rather than environmental factors. Whereas the common polygenic obesity arises when an individual genetic make-up is susceptible to an environment that promotes energy consumption over energy expenditure, monogenic obesity, on the contrary, is the obesity associated with a single gene mutation, which is sufficient by itself to cause weight gain in a food abundant context. Genes involved in the leptin-melanocortin pathway are often mutated in these cases. The cumulative prevalence of monogenic obesity among children with severe obesity is about 5%.

Recently, deletions in the region p11.2 of the chromosome 16 encompassing the gene SH2B1, which is involved in the leptin and insulin signaling, have been reported in about 0.5% of children with severe early-onset obesity. These patients show extreme hyperphagia, severe insulin resistance and, in some cases, mild developmental delay.

## Introduction

Childhood obesity appears particularly alarming considering the strong association with increased rates of premature death from endogenous causes observed in a cohort of American children followed for more than 20 years [1].

Ipercaloric diet and reduced physical activity have driven the rise in the prevalence of adult and childhood obesity over a relatively short time interval [2,3]. The genetic contribution to body weight has been established through family studies investigating parent-offspring relationships and the study of twins and adopted children. These studies have led to the conclusion that the strong predictive value of parental body mass index (BMI) mainly stems from genetic rather than environmental factors. Twin studies, in comparison to family and adoption studies, have revealed that between 40 and 70% of the BMI variance within a population can be expalined by genetic effects [4-6].

The molecular approach has revealed several candidate genes for the human obesity and has cleary positioned interactions between genetic make-up and enviroment to understand the mechanisms involved in fat-mass expansion [7-9].

Considering the genetic point of view obesity is classified in i) monogenic obesity, that is the obesity associated with a single gene mutation; in these cases single gene variants are sufficient by themselves to cause obesity in food abundant societies; patients with monogenic obesity usually show extremely severe phenotypes characterized by childhood obesity onset, often associated with additional behavioral, developmental or endocrine disorders [10,11]; ii) syndromic obesity (this includes some Mendelian disorders in wich patients are clinically obese and are additionally distinguished by mental retardation, dysmorfic features, and organ-specific developmental abnormalities) [12] and iii) polygenic obesity; this very common kind of obesity, which concerns the great majority of obese children, arises when an individual genetic make-up is susceptible to an environment that promotes energy consumption over energy expenditure [13].

The study of extreme human obesity caused by single gene defects has provided a glimpse into the long-term regulation of body weight and has shown that the hypothalamic leptin-melanocortin system is critical for energy balance in humans, because disruption of this pathway causes the most severe obesity phenotypes. Briefly, leptin is an adipocyte-derived satiety hormone that signals the size of the fat depot interacting with the leptin receptor in the nucleus arcuatus of the hypothalamus. The signal is then forwarded via the alpha-melanocyte stimulating hormone to the melanocortin-4 receptor allowing appetite suppression and increasing energy expenditure. Mutations in genes involved in this pathway accounts for not more than 5% of all childhood severe obesity cases (Table 1). In fact, whereas mutations of the leptin (LEP) and proopiomelanocortin (POMC) genes are rare [14,15], both mutations of the melanocortin-4 receptor (MC4R) and of the leptin receptor (LEPR) genes have been reported in about 2-3% of the children with severe obesity [16,17].

**Table 1:** Childhood early-onset monogenic obesity due to the involvement of the genes belonging to the leptin-melanocortin pathway.

GENE	CHROMOSOME	INHERITANCE	PREVALENCE	PHENOTYPIC FEATURES
LEP	7q31.1	Recessive	Rare	Severe obesity, Hyperphagia, Hypogonadotropic hypogonadism, Central hypothyroidism.

LEPR	1p31	Recessive	1.5%	Severe obesity, Hyperphagia, Hypogonadotropic hypogonadism and central hypothyroidism (in some cases)

POMC	2p23.3	Recessive	Rare	Severe obesity, Hyperphagia, Hypothyroidism, ACTH deficiency, Red hair (among Caucasians), Increased growth.

MC4R	18q22	Dominant	2.5%	Severe obesity, Hyperphagia, Excess fat and lean mass, Severe hyperinsulinaemia, Increased growth.

Deletions involving SH2B1	16p11.2	Dominant or de novo	0.5%	Severe obesity, Hyperphagia and severe insulin resistance disproportionate for the degree of obesity, Mild developmental delay (in some cases).

## Discussion

In the last years it has been discovered that genetic differences among people can derive from lost or duplicated segments of chromosomes, called copy number variants (CNVs) [18].

A recent work of Farooqi et al analyzed the genomes of 300 obese children for missing or duplicated chromosome segments [19]. All patients had severe obesity defined as a BMI standard deviation score >3, obesity onset before 10 years of age and 143 of them also showed developmental delay. Mutations in LEPR, POMC and MC4R genes were previously excluded by direct nucleotide sequencing. A number of CNVs were more common in the obese children than in a group of normal-weight controls (p = 0.0005). The commonest CNV found in these patients was identified in five unrelated children harbouring overlapping deletions on chromosome 16p11.2. In three patients this deletion co-segregated with severe obesity, whereas in the remaining two children the deletion occurred *de novo *and was particularly large, extending through a region previously associated with autism and mental retardation. Interestingly, both patients had mild developmental delay in addition to severe obesity. Pooling the data obtained in a replication cohort of 1062 Caucasian obese patients with those from the original discovery set, the prevalence of chromosome 16p11.2 deletions associated to severe early-onset obesity alone (i.e.; without developmental delay) was 0.41%. The same kind of deletion was found in only 2 out of 7366 controls (p < 0.001).

All these deletions encompass several genes, some of them involved in neurological disorders or immunity, but include also the gene SH2B1, which is known to be involved in insulin signaling [20]. Mice lacking this gene become extremely fat and develop hyperphagia, insulin resistance, hepatic steatosis and type 2 diabetes [21,22]. Furthermore, SH2B1 is a key intermediary in leptin signaling, promoting the activation of the leptin signaling pathway downstream of JAK2 [23-25]. Additionally, neuron-specific overexpression of SH2B1 in obese mice, protects against diet-induced leptin and insulin resistance [26].

Accordingly, the phenotype of the children with SH2B1-containing deletions is characterized by extreme hyperphagia and fasting insulin levels disproportionately elevated compared to age and obesity-matched controls [19].

Similar data (i.e.; deletions on chromosome 16p11.2) have been reported in a population prevalently made up by adults with severe obesity, further demonstrating the potential importance in common diseases of rare variants with strong effects [27].

## Conclusions

The progress in the knowledge of the human genome and the discovery of new genetic disorders allowed to categorize the human obesity as a medical condition rather than simply a moral failing.

The clinical evaluation of the severely obese children is becoming increasingly sophisticated. There are a lot of genes that can be implicated in the obesity and obese patients in the world are too much to perform molecular study for everyone. It would be useful to identify particular phenotypes and clinical features that can help to recognize the subjects to investigate with genetic screening. The presence of severe obesity in a young child (<5 yr old) associated to extreme hyperphagia, severe insulin resistance disproportionate for the degree of obesity and a positive family history of early-onset obesity may support a genetic analysis. The cumulative prevalence of monogenic obesity among children with severe obesity is not more than 5% (Table 1). Nevertheless, considering the great proportion of obese children worldwide [28], the possibility exists that in countries such as Italy, many thousands of children might be carriers of this kind of mutations.

In conclusion, the work of Farooqi et al reports the first rare CNVs associated with obesity, suggests a role for SH2BI in human energy homeostasis and glucose metabolism and adds another important contribution to the understanding of the complicated puzzle of the genetic basis of early onset obesity.

## Competing interests

The authors declare that they have no competing interests.

## Authors' contributions

LP, PM and AG were involved in drafting the manuscript, EMdG revised it critically. All authors read and approved the final manuscript.
